# Predicting outcomes of continuous renal replacement therapy using body composition monitoring: a deep-learning approach

**DOI:** 10.1038/s41598-023-30074-4

**Published:** 2023-03-21

**Authors:** Kyung Don Yoo, Junhyug Noh, Wonho Bae, Jung Nam An, Hyung Jung Oh, Harin Rhee, Eun Young Seong, Seon Ha Baek, Shin Young Ahn, Jang-Hee Cho, Dong Ki Kim, Dong-Ryeol Ryu, Sejoong Kim, Chun Soo Lim, Jung Pyo Lee, Korean Association for the Study of Renal Anemia and Artificial Intelligence (KARAI), Sung Gyun Kim, Gang Jee Ko, Jung Tak Park, Tae Ik Chang, Sungjin Chung, Jung Pyo Lee, Sang Ho Lee, Bum Soon Choi, Jin Seok Jeon, Sangheon Song, Dae Eun Choi, Woo Kyung Jung

**Affiliations:** 1 Division of Nephrology, Department of Internal Medicine, Ulsan University Hospital, University of Ulsan College of Medicine, Ulsan, Republic of Korea; 2 Lawrence Livermore National Laboratory, Livermore, CA USA; 3 University of British Columbia, Vancouver, Canada; 4 Division of Nephrology, Department of Internal Medicine, Hallym University Sacred Heart Hospital, Anyang, Republic of Korea; 5 Division of Nephrology, Department of Internal Medicine, Sheikh Khalifa Specialty Hospital, Ra’s al Khaimah, United Arab Emirates; 6 Division of Nephrology, Department of Internal Medicine, Pusan National University Hospital, Busan, Republic of Korea; 7 Division of Nephrology, Department of Internal Medicine, Hallym University Dongtan Sacred Heart Hospital, Hwaseong, Republic of Korea; 8 Division of Nephrology, Department of Internal Medicine, Korea University Guro Hospital, Seoul, Republic of Korea; 9 Division of Nephrology, Department of Internal Medicine, Kyungpook National University Hospital, Daegu, Republic of Korea; 10 Division of Nephrology, Department of Internal Medicine, Seoul National University Hospital, Seoul, Republic of Korea; 11 Department of Internal Medicine, Seoul National University College of Medicine, Seoul, Korea; 12 Kidney Research Institute, Seoul National University College of Medicine, Seoul, Korea; 13 Division of Nephrology, Department of Internal Medicine, School of Medicine, Ehwa Womans University, Seoul, Republic of Korea; 14 Division of Nephrology, Department of Internal Medicine, Seoul National University Bundang Hospital, Seongnam, Republic of Korea; 15 Center for Artificial Intelligence in Healthcare, Seoul National University Bundang Hospital, Seongnam, Republic of Korea; 16 Division of Nephrology, Department of Internal Medicine, Seoul National University Boramae Medical Center, 20 Boramae-Ro 5-Gil, Dongjak-gu, Seoul, 156-707 Republic of Korea; 17 Korean Association for the Study of Renal Anemia and Artificial Intelligence (KARAI), Anyang, Republic of Korea; 18 Seoul National University College of Medicine, Seoul, Korea; 19 Hallym University College of Medicine, Anyang, Korea; 20 Korea University College of Medicine, Seoul, Korea; 21 Yonsei University College of Medicine, Seoul, Korea; 22 National Health Insurance Service Ilsan Hospital, Goyang, Korea; 23 College of Medicine, The Catholic University of Korea, Seoul, Korea; 24 College of Medicine, Kyung Hee University, Seoul, Korea; 25 Soon Chun Hyang University, Seoul, Korea; 26 Pusan National University College of Medicine, Busan, Korea; 27 Chungnam National University College of Medicine, Daejeon, Korea; 28 Gachon University of Medicine and Science, Incheon, Korea

**Keywords:** Renal replacement therapy, Biotechnology

## Abstract

Fluid balance is a critical prognostic factor for patients with severe acute kidney injury (AKI) requiring continuous renal replacement therapy (CRRT). This study evaluated whether repeated fluid balance monitoring could improve prognosis in this clinical population. This was a multicenter retrospective study that included 784 patients (mean age, 67.8 years; males, 66.4%) with severe AKI requiring CRRT during 2017–2019 who were treated in eight tertiary hospitals in Korea. Sequential changes in total body water were compared between patients who died (event group) and those who survived (control group) using mixed-effects linear regression analyses. The performance of various machine learning methods, including recurrent neural networks, was compared to that of existing prognostic clinical scores. After adjusting for confounding factors, a marginal benefit of fluid balance was identified for the control group compared to that for the event group (*p* = 0.074). The deep-learning model using a recurrent neural network with an autoencoder and including fluid balance monitoring provided the best differentiation between the groups (area under the curve, 0.793) compared to 0.604 and 0.606 for SOFA and APACHE II scores, respectively. Our prognostic, deep-learning model underlines the importance of fluid balance monitoring for prognosis assessment among patients receiving CRRT.

## Introduction

For patients with severe acute kidney injury (AKI) admitted to the intensive care unit (ICU), continuous renal replacement therapy (CRRT) is recommended according to their clinical condition. In Korea, the number of patients on CRRT for AKI has increased rapidly, from 4,667 patients in 2005–2007 to 13,414 patients in 2014–2016^1^. Severe AKI requiring CRRT increases the risk of mortality and is also a significant medical concern because of its impact on the incidence of cardiovascular disease (CVD) and chronic kidney disease (CKD)^2^. The epidemiology and outcomes of AKI across multiple cohorts have been reported by Hostes et al.^3^: renal replacement therapy (RRT) has been found to be required in 15% of patients with septic AKI and in 5–11% of non-septic patients in the ICU. Moreover, as more than one-third of patients with AKI admitted to the ICU require CRRT, management of AKI is a significant challenge for nephrologists and intensivists^4^.

The mechanisms underlying the progression from pre-renal AKI to AKI have been studied, and early supportive fluid therapy has been recognized to improve outcomes using various automated methods^5–7^. Notably, a deep-learning approach using information extracted from electronic health records has accurately predicted the development of AKI among in-hospital patients^7^. To date, however, there is a paucity of accurate prediction models for patients with AKI and a poor prognosis who require CRRT. Machine learning (ML) has been extensively tested in the field of kidney disease, although ML approaches to predict prognosis among patients undergoing CRRT are limited^8,9^. The technological purpose of deep learning is not to perform a comprehensive statistical analysis of existing data but, rather it is to increase the capacity to forecast the necessary acquisition of future data^10^. Deep-learning algorithms allow complex nonlinear patterns to be detected in a high-dimensional space, which cannot be easily achieved using standard ML algorithms. To our knowledge, deep learning has not been used to study cases of AKI requiring CRRT. Although the interpretability and explainability of current deep-learning algorithms are limited compared to traditional statistical methods, deep-learning algorithms can predict the realization of dependent variables more accurately on unseen data than traditional methods. Hence, we employed deep-learning models in this study to improve therapeutic considerations instead of building complex and novel artificial intelligence (AI) technologies^6,11^. Previous prognosis prediction studies have adequately shown that AI technology may be used to predict the risk of AKI using well-known technology^6,11^. However, identifying instances of significant AKI progression among patients requiring CRRT remains difficult^6,11^ as the critical issue of fluid balance in these patients has not been accurately addressed^12,13^. Precision CRRT allows the adjustment of the ultrafiltration rate and body composition monitoring (BCM) based on each patient’s phenotype. Accordingly, clinical outcomes may be improved in certain populations, allowing for individualized therapy based on each patient’s volume status^13–16^.

Excessive volume loss may precipitate hypotension and impair renal survival, resulting in reduced RRT-free survival^12^. Excessive volume overload may exacerbate pulmonary congestion, resulting in reduced ventilator-free survival^12^. Numerous studies have demonstrated an association between fluid overload and poor clinical outcomes in critically ill patients^17,18^ and patients with severe AKI^19^. Elevated cumulative fluid balance during the initial 72 h of ICU admission increases the risk of in-hospital mortality in patients with septic AKI^20^. Nevertheless, a negative daily fluid balance has been consistently associated with superior clinical outcomes^17,21^. However, the physical measures of fluid balance utilized in these studies have been inconsistent and based only on body weight measurements. Therefore, in this study, we aimed to determine whether repeated recording of fluid balance, using BCM values, could improve prognostic prediction using various ML methods, including deep-learning techniques, such as recurrent neural networks (RNNs), for patients with AKI requiring CRRT.

## Results

### Baseline characteristics

**Figure 1:**
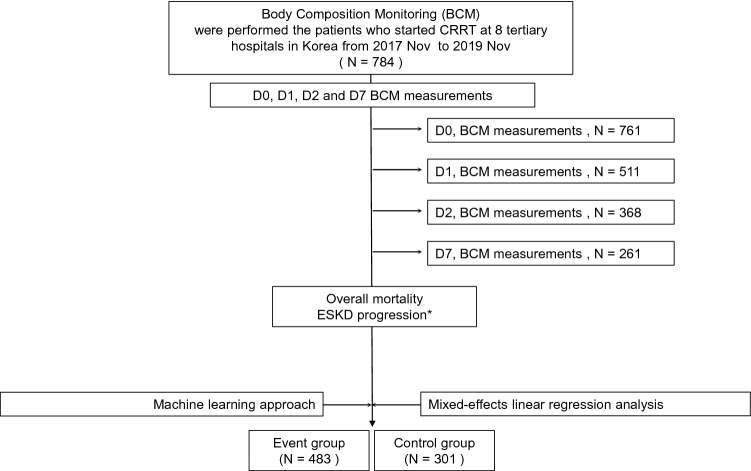
Study flowchart. End-stage kidney disease is defined by the maintenance of dialysis at 3 months after CRRT initiation. Body composition monitoring is performed using InBody.

After selection, 784 patients were included in the final analysis (Fig. 1). Table 1 presents patient information. The mortality rate was 61.6% (483 patients) overall. End-stage renal disease, defined as the continued need for RRT at 3 months after CRRT initiation, was identified in 77 (9.8%) patients. There was no significant difference in baseline variables, such as age, sex, and age-modified Charlson Comorbidity Index (CCI), between the event and control groups. Sepsis was the most frequently reported cause of AKI in both groups, with a higher prevalence in the event group (57.6%) than in the control group (47.5%; *p* = 0.06).

**Table 1:** Patient baseline characteristics at the time of CRRT initiation.

	Event (death) (n = 483)	Control (alive) (n = 301)	*P* value
Male sex (n, %)	315 (65.2)	206 (68.4)	0.353
Age, mean (SD), years	68.4 ± 13.9	66.7 ± 14.4	0.110
Body mass index, mean (± SD), kg/m^2^	23.5 ± 4.0	24.2 ± 3.9	0.024
Preexisting conditions (n, %)			
Hypertension	222 (46.0)	177 (58.8)	< 0.001
Diabetes mellitus	185 (38.3)	162 (53.8)	< 0.001
Coronary artery disease	33 (6.8)	16 (5.3)	0.394
Heart failure	50 (10.4)	41 (13.6)	0.165
Liver disease	158 (32.7)	81 (26.9)	0.086
Cancer	157 (32.5)	49 (16.3)	< 0.001
Age-modified CCI, mean (SD)	3.2 ± 2.2	3.0 ± 1.9	0.363
Contributing factors for AKI (n, %)			
Sepsis	278 (57.6)	143 (47.5)	0.006
Ischemia	69 (14.3)	58 (19.3)	0.065
Major surgery	33 (6.8)	23 (7.6)	0.669
Nephrotoxic event	24 (5.0)	26 (8.6)	0.041
Cardiogenic event	12 (2.5)	6 (2.0)	0.655
Laboratory findings at ICU admission, mean (SD)			
Hemoglobin	9.8 ± 2.4	10.0 ± 2.3	0.377
WBC	15,123 ± 12,015	14,584 ± 9427	0.508
PLT	123,864 ± 98,450	150,953 ± 100,305	< 0.001
Serum creatinine, mg/dL	3.0 ± 1.8	4.2 ± 2.9	< 0.001
MDRD eGFR	25.8 ± 17.6	21.6 ± 19.8	0.003
Sodium, mEq/L	136.9 ± 7.3	136.5 ± 6.2	0.403
Potassium, mEq/L	4.5 ± 1.0	4.4 ± 1.0	0.296
Ca	7.8 ± 1.1	8.0 ± 1.2	0.170
P	5.5 ± 2.6	5.1 ± 2.2	0.012
Albumin, mg/dL	2.6 ± 0.5	2.8 ± 0.5	< 0.001
Bilirubin	3.5 ± 5.6	1.8 ± 3.3	< 0.001
AST, U/L	912.4 ± 2187.5	705.2 ± 2179.1	0.199
ALT, U/L	325.4 ± 757.3	309.8 ± 958.9	0.802
PT INR	1.9 ± 1.2	1.6 ± 0.8	< 0.001
pH	7.31 ± 0.12	7.34 ± 0.11	0.001
Lactate	8.2 ± 14.6	6.0 ± 16.6	0.091
CRP	24.2 ± 64.2	20.2 ± 42.7	0.382
CRRT setting			
Prescribed dose, mean (SD), mL/kg/h	38.5 ± 14.4	38.2 ± 13.3	0.766
Delivered dose, mean (SD), mL/kg/h	34.3 ± 8.6	33.7 ± 8.7	0.358

Continuous variables are presented as means (standard deviations) and categorical variables as frequencies (percentages). *SD* standard deviation, *CCI* comprehensive complication index, *AKI* acute kidney injury, *ICU* intensive care unit, *WBC* white blood count, *PLT* platelet level, *MDRD* modification of diet in renal disease, *AST* aspartate aminotransferase, *ALT* alanine aminotransferase, *PT* prothrombin time, *INR* international normalized ratio, *CRP* C-reactive protein, *CRRT* continuous renal replacement therapy.

Clinical and biochemical data were obtained at the time of CRRT initiation. There were no between-group differences in hemoglobin level, leukocyte count (white blood cell count), electrolyte (Na, K, and Ca) level, and C-reactive protein level. However, serum albumin level and blood venous pH were significantly lower in the event group than in the control group (Table 1). The mean delivered CRRT dose was 34.3 ± 8.6 mL/kg/h in the event group and 33.7 ± 8.7 mL/kg/h in the control group (*p* = 0.358).

### Clinical parameters and volume status assessments during CRRT

Clinical and biochemical volume status assessments, using BCM, are presented in Tables 2 and 3. There were no between-group difference in urine output for 2 h and 6 h after CRRT initiation. However, the clinical severity indexes and Acute Physiology and Chronic Health Evaluation II (APACHE) II, Sequential Organ Failure Assessment (SOFA), and Glasgow Coma Scale (GCS) scores were relatively higher in the event group than in the control group. The mean APACHE II score was higher in the event group than in the control group (29.2 ± 10.3 vs. 26.5 ± 9.0; *p* < 0.001). Patients who died while receiving CRRT had lower systolic blood pressure, lower mean arterial pressure, and a greater rate of vasopressor use at the time of CRRT initiation. Mechanical ventilation was used to a greater extent in the event group than in the control group (Table 2). There were no between-group differences in volume status, as assessed by body weight and BCM, at baseline (day 0) and no between-group differences in body weight at days 1, 2, and 7 after CRRT initiation. Notably, the total body water/height^2^ (TBW/H^2^) level was higher in the event group than in the control group, at day 2 (13.9 ± 2.7 L/m^2^ vs. 13.4 ± 2.3 L/m^2^; *p* = 0.071). Seven days following CRRT initiation, the TBW/H^2^ level was 13.3 ± 2.5 L/m^2^ in the event group and 13.2 ± 1.9 L/m^2^ in the control group (*p* = 0.669; Table 2).

**Table 2:** Clinical parameters at the time of CRRT initiation.

	Event (death) (n = 483)	Control (alive) (n = 301)	*P* value
SBP (mmHg)	112.7 ± 23.3	120.4 ± 26.3	< 0.001
DBP (mmHg)	64.4 ± 15.3	65.2 ± 15.4	0.507
MAP (mmHg)	79.1 ± 16.1	81.9 ± 17.6	0.020
APACHE II score, mean (SD)	29.2 ± 10.3	26.5 ± 9.0	< 0.001
SOFA score, mean (SD)	10.8 ± 3.7	9.7 ± 3.5	< 0.001
GCS score, mean (SD)	7.5 ± 4.5	9.0 ± 4.6	< 0.001
Mechanical ventilation (n, %)	329 (68.1)	174 (57.8)	0.003
Vasopressor support (n, %)	373 (77.2)	200 (66.4)	0.001
2-h U/O before CRRT, mean (SD), mL	75.0 ± 120.7	76.5 ± 135.6	0.872
6-h U/O before CRRT, mean (SD), mL	208.1 ± 317.0	199.4 ± 293.5	0.717
24-h total U/O, mean (SD), mL	698.1 ± 820.8	547.0 ± 710.5	0.014

Continuous variables are presented as means (standard deviations), and categorical variables are presented as frequencies (percentages). *SBP* systolic blood pressure, *DBP* diastolic blood pressure, *MAP* mean arterial pressure, *APACHE II* acute physiology and chronic health evaluation II, *SOFA* sequential organ failure assessment, *GCS* glasgow coma scale, *CRRT* continuous renal replacement therapy, *U/O* urinary output, *SD* standard deviation.

**Table 3:** Volume status assessments using BCM during CRRT.

	Event (death) (n = 483)	Control (alive) (n = 301)	*P*-value
Body weight at day 0 before CRRT	63.2 ± 12.4	64.5 ± 12.7	0.171
TBW/H^2^ at day 0 before CRRT initiation (D0) mean (SD), L/m^2^	13.5 ± 2.6	13.4 ± 2.3	0.563
Body weight at day 1	64.6 ± 12.7	65.0 ± 12.4	0.737
TBW/H^2^ at day 1	14.0 ± 2.5	13.6 ± 2.2	0.111
Body weight at day 2	63.9 ± 13.1	64.3 ± 12.8	0.739
TBW/H^2^ at day 2	13.9 ± 2.7	13.4 ± 2.3	0.071
Body weight at day 7	62.3 ± 12.0	62.8 ± 12.0	0.717
TBW/H^2^ at day 7	13.3 ± 2.5	13.2 ± 1.9	0.669

Continuous variables are presented as means (standard deviations) and categorical variables as frequencies (percentages). CRRT, continuous renal replacement therapy; TBW/H^2^, total body water/height^2^ (L/m^2^).

### Modeling process

**Figure 2:**
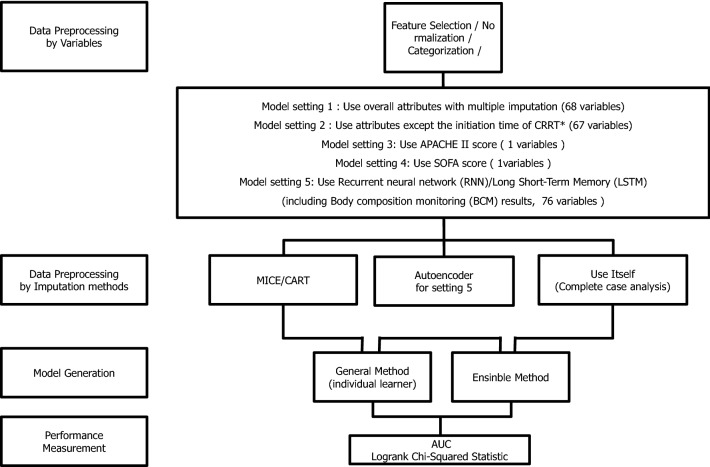
Model structure.

For the initial implementation of ML, we divided the model approach into five settings (Fig. 2). Model setting 1 involved the application of global characteristics through multiple imputations using 68 variables, whereas model setting 2 included all characteristics, with the exception of the CRRT * startup time from the time of ICU admission, and, thus, did not capture early CRRT initiations; instead, CRRT was entered as a continuous variable. Model setting 3 only used the APACHE II score, and model setting 4 only used the SOFA score. Finally, in model setting 5, an RNN and long short-term memory (LSTM) network were employed to examine all 76 variables collected, including the BCM data.

### Between-group comparison of changes in TBW/H^2^ and body weight

**Figure 3:**
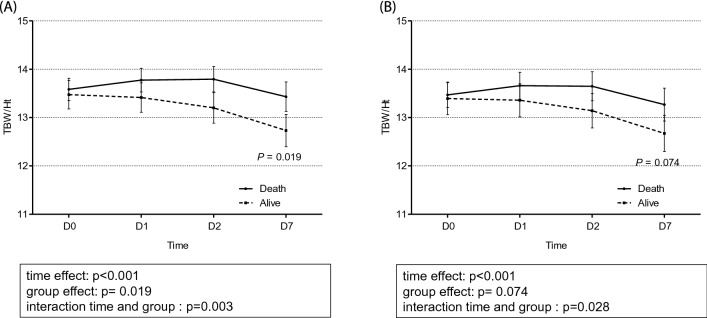
Comparison of the change in TBW/H^2^ estimates between the mortality and alive groups. (**A**) Univariate and (**B**) multivariate mixed-effect linear regression analyses are shown. The mixed-effects linear regression analysis between the two groups included the association with volume status at CRRT initiation, adjusted for age, sex, age-modified CCI, DM, hypertension, SOFA score, urine output for 6 h, and serum albumin level, MDRD, and eGFR at CRRT initiation.

**Figure 4:**
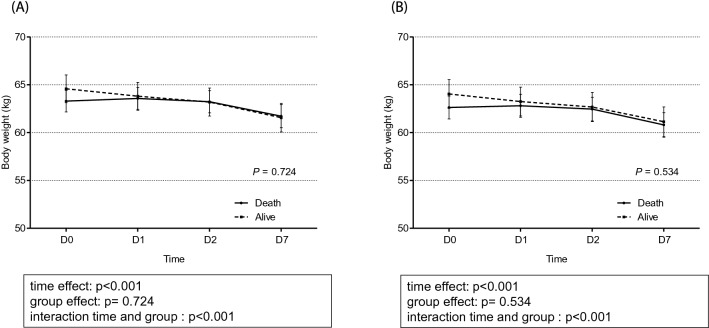
Comparison of the change in body weight estimates between the mortality and alive groups. (**A**) Univariate and (**B**) multivariate mixed-effect linear regression analyses are shown. The mixed-effects linear regression analysis between the two groups included the association with volume status at CRRT initiation, adjusted for age, sex, age-modified CCI, DM, hypertension, SOFA score, urine output for 6 h, and serum albumin level, MDRD, and eGFR at CRRT initiation.

**Figure 5:**
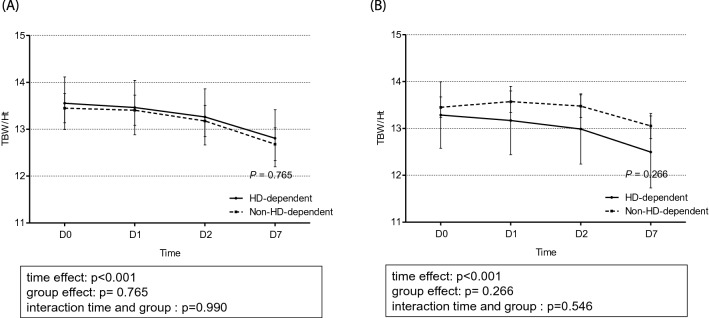
Comparison of the change in TBW/H^2^ estimates between the dialysis dependent group and non-dialysis dependent groups. (**A**) Univariate and (**B**) multivariate mixed-effect linear regression analyses are shown. The mixed-effects linear regression analysis between the two groups included the association with volume status at CRRT initiation, adjusted for age, sex, age-modified CCI, DM, hypertension, SOFA score, urine output for 6 h, and serum albumin level, MDRD, and eGFR at CRRT initiation.

**Figure 6:**
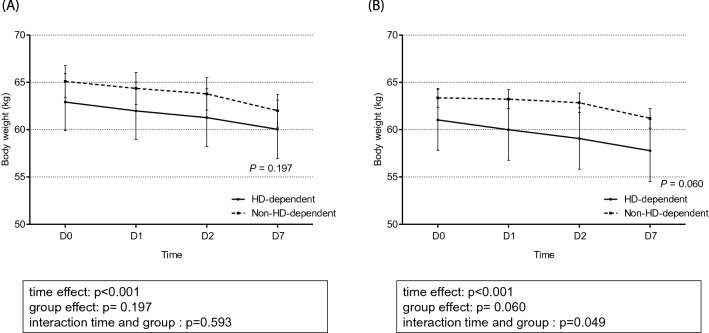
Comparison of the change in body weight estimates between the dialysis dependent group and non-dialysis dependent groups. (**A**) Univariate and (**B**) multivariate mixed-effect linear regression analyses are shown. The mixed-effects linear regression analysis between the two groups included the association with volume status at CRRT initiation, adjusted for age, sex, age-modified CCI, DM, hypertension, SOFA score, urine output for 6 h, and serum albumin level, MDRD, and eGFR at CRRT initiation.

The total TBW/H^2^ level was higher in the event group than in the control group (Fig. 3). The changes in the TBW/H^2^ level over time were considerably different between the two groups (*p* < 0.001). The linear mixed model analysis revealed a time-dependent increase in between-group differences in TBW/H^2^, although this difference was not significant (*p* = 0.074) after adjusting for confounding factors of age, sex, age-modified CCI, history of diabetes mellitus, hypertension, SOFA score, urine output for 6 h, serum albumin, and the Modification of Diet in Renal Disease equation, which estimates the glomerular filtration rate at CRRT initiation. Specifically, in the control group, the TBW/H^2^ level steadily dropped from 13.39 ± 0.16 L/m^2^ at day 0 to 12.67 ± 0.19 L/m^2^ at day 7, while in the event group, the TBW/H^2^ remained steadily high from day 0 (13.47 ± 0.13 L/m^2^) to day 7 (13.26 ± 0.17 L/m^2^), as shown in Fig. 3. With regard to change in body weight estimates, the linear mixed-effect model analysis did not reveal a between-group difference (*p* = 0.534), despite a significant time-dependent effect on body weight in both groups (*p* < 0.001; Fig. 4). A comparison of the changes in TBW/H^2^ and body weight between HD-dependent and non-HD-dependent patients on CRRT is shown in Fig. 5. Interestingly, the linear mixed model analysis revealed a substantial difference in body weight change over time between HD-dependent and non-HD-dependent patients (*p* = 0.060; Fig. 6). In the HD-dependent group, body weight declined quickly over time, from 61.02 ± 1.63 kg at day 0 to 59.98 ± 1.64 kg at day 1, 59.06 ± 1.65 kg at day 2, and 57.77 ± 1.67 kg at day 7. By contrast, the decline in body weight was modest in the non-HD-dependent group, from 63.35 ± 0.50 kg at day 0 to 63.22 ± 0.50 kg at day 1, 62.83 ± 0.51 kg at day 2, and 61.19 ± 0.53 kg at day 7.

### Comparison of the classification model for mortality (event group) using conventional algorithm and severity scores

The findings regarding the model parameters used in this study, including the imputation technique, validation method and ratio, size of the test set, and performance of the test set are shown in Fig. 2 and Table 4. The performance of the ML method for classification was compared with various parameters using the area under the curve (AUC) according to the model setting (Fig. 2; Table 4). We separated model settings 1 and 2 to evaluate whether the prognostic predictive power would be changeable if the early start of the CRRT treatment regimen was included as a variable. The overall attributes with multiple imputations (68 variables), including the initiation time of CRRT, of setting 1 are presented in the Supplemental Table S2. The overall attributes with multiple imputations (67 variables), excluding the initiation time of CRRT, of setting 2 are presented in Table 2. The random forest (AUC, 0.7678) and bagging (AUC, 0.7494) models with cross-validation, which were built from the dataset using the ensemble technique, were found to have enhanced predictive performance compared to the logistic regression analysis (AUC, 0.6770) and APACHE II (AUC, 0.5646) and SOFA (AUC, 0.6505) scoring systems.

**Table 4:** Classification model for mortality using the conventional algorithm.

Setting	Validation method	Test set size	Independence variables	Model	Test performance (AUC)
2	Cross-validation	234	67	Random forest	0.7678
2	One validation set	234	67	Random forest	0.7606
2	Cross-validation	234	67	Bagging	0.7494
2	One validation set	234	67	Bagging	0.7250
2	Cross-validation	234	67	Lasso	0.7199
2	Cross-validation	234	67	Ridge	0.7188
2	One validation set	234	67	Ridge	0.7082
2	One validation set	234	67	Lasso	0.7057
2		234	67	Logistic regression	0.6754
2	One validation set	234	67	Decision tree	0.6696
2	Cross-validation	234	67	Decision tree	0.6271
3		234	1	APACHE II score	0.5646
4		234	1	SOFA score	0.6505

*AUC* area under the curve, *APACHE II* acute physiology and chronic health evaluation II, *SOFA* sequential organ failure assessment.

### Comparison of the classification model for mortality (event group) using the deep-learning model with RNN

We performed an additional analysis to apply a deep-learning algorithm using longitudinal data to evaluate further enhancement of the prediction model. The mortality risk model was validated using deep neural network algorithms and compared it with conventional algorithms. Our proposed deep-learning model comprised LSTM networks and an autoencoder. The former was introduced to deal with time-series data, whereas the latter was used to compensate for missing data. Figure 2 demonstrates the analyzed records for model setting 5, which used 76 independent attributes to guide learning, including repeated fluid balance data and BCM results. Among the various conventional algorithms used, the RNN with LSTM networks model yielded the highest AUC value (0.7938), with inclusion of an autoenconder using 68 variables (setting 1) yielding an AUC value of 0.7912 (Table 5).

**Table 5:** Classification model for mortality using the deep-learning model with the recurrent neural network.

Test set size	Independence variables	Imputation method	Model	Test performance (AUC)
192	76	Autoencoder	RNN/LSTM	0.7938
192	68	Autoencoder	Vanilla RNN	0.7912
192	68	MICE/CART	Random forest	0.7875
192	68	MICE/CART	Vanilla RNN	0.7686
192	68	MICE/CART	Bagging	0.7652
192	68	MICE/CART	Lasso	0.7143
192	68	MICE/CART	Ridge	0.7115
192	68	MICE/CART	Logistic regression	0.7079
192	68	MICE/CART	Decision tree	0.6273
192	1		APACHE II	0.6063
192	1		SOFA	0.6040

*AUC* area under the curve, *MICE* multivariate imputation by chained equations, *CART* classification and regression trees, *RNN* recurrent neural network, *LSTM* long short-term memory, *APACHE II* acute physiology and chronic health evaluation II, *SOFA* sequential organ failure assessment.

## Discussion

The main finding of our study is that repeated monitoring of fluid balance among patients with AKI requiring CRRT in the ICU can improve the predictability of prognosis. Our findings, based on a retrospective analysis of a multicenter prospective cohort, underlines the significance of repeated BCM using BIA as a treatment guide for the aforementioned clinical population, with excess body water possibly being an independent risk factor for death, as determined using conventional analysis with a linear mixed-effect model. We further demonstrated that the inclusion of repeated measurements of BCM in a deep-learning approach better predicted mortality than classical ML methods.

Optima fluid status is pivotal, and the concept of “dry weight” is an established performance to guide HD for patients with end-stage kidney disease (ESKD). However, as sustained euvolemia is difficult to achieve in patients without residual kidney function, various efforts have been made to enhance the accuracy of dry weight estimates to guide HD. Randomized controlled studies have provided evidence for the combined use of BIA with other markers to assess weight gain in patients undergoing peritoneal dialysis^22^ and chronic HD^23^. Three meta-analyses of RCTs found BIA-guided therapy to be somewhat beneficial in controlling blood pressure and left ventricular hypertrophy among patients on HD, including peritoneal dialysis, but not for improving overall survival^24–26^. Consequently, whether further BIA measures would improve clinical outcomes in patients with severe AKI requiring CRRT has been raised as a clinical issue of interest. Only a few studies conducted in the recent past have shown that BIA is effective in determining the volume status of critically ill patients in the ICU^27^. The InBody S10 (InBody, Seoul, Korea)^28^, a body composition analyzer, has touch-type or adhesive electrodes, and is intended for patients who are immobile or amputees necessitating BIA. In this study, BIA was performed repeatedly using InBody, which has been validated to allow for body composition monitoring in the Korean surgical ICU after major surgery^29^, predicting the severity of patient condition in surgical ICU^30^. It has also been revealed to be not inferior to CT for the measurement of muscle mass^31^. At first, in this study, using the linear mixed-effect model approach, we first determined whether the BIA values of the non-survivor (event) and survivor (control) groups were different by repeating BIA measurements. In the univariate analysis, TBW/H^2^ values revealed a significant effect of time between groups (*p* = 0.003), with positive fluid balance being higher in the event group than in the control group after adjusting for covariates, although this difference was not significant (*p* = 0.074; Fig. 2). Interestingly, despite the protocol for fluid balancing therapy used, a significant time-dependent effect on body weight control was observed in both groups, with no between-group difference (*p* = 0.534; Fig. 3). Our findings are important in this regard as volume status might be difficult to evaluate from body weight alone. Our results show that repeated BCM monitoring and guiding the treatment process for CRRT may provide a more accurate estimate of fluid overload than body weight and, thus, provide improved treatment outcomes. These results are corroborated by our finding that, at 3 months after CRRT initiation, a substantially lower retained body weight was associated with a higher risk of dialysis-dependent status. A previous study using BIA^23^, in which residual renal function was defined by urine volume, reported a considerable increase in the risk of anuria with a substantial decrease in body weight among patients on HD. In addition, our findings indicate that the use of the TBW level, which is concurrent with body weight, may be preferable to prevent excessive ultrafiltration and a resulting decrease in residual renal function.

Although ML has been widely studied in the context of patients admitted to the ICU, little is known regarding the use of ML methods for patients with CCRT^8,9^. Considering the heterogenicity and medical severity of patients requiring CCRT^12,13^, various critical patient prognostic scoring systems have been introduced, even before ML was introduced, with previous attempts to augment the prognostic value of scoring systems, including the SOFA and APACHE scores, using deep learning^32^. Although these existing scoring systems indicate systemic disease severity among patients in the ICU, they do not include changes in fluid dynamics, which are critical for patients with severe AKI. Monitoring fluid balance status is specifically important for CRRT, which is used for organ support in patients presenting with multi-organ failure, unlike maintenance HD, which is designed as a chronic RRT^33,34^. Body fluid balance, which reflects the systemic disease state, is a significant clinical variable related to the expansion of extracellular fluid balance and lung congestion; as such, it is an important predictor of death not only for patients with severe AKI^19^ but also for patients with CKD^20^. Several studies have tried to overcome the problem of fluid accumulation in pulmonary conditions related to ventilator-free survival and mortality among patients receiving ventilator support using ML^35,36^. However, in both of the abovementioned studies, only 10%^35^ and 14%^36^ of patients with renal failure requiring RRT were included, respectively. By comparison, 62% of patients in our study cohort were on ventilator support, with our findings indicating that fluid balance may be an essential prognostic variable in this clinical population. This is the reason we chose setting 5 in our deep-learning model, which included BCM data, with this setting improving the predictive performance of the model (AUC 0.7938).

Euvolemia is a critical goal of CRRT in both patients on maintenance dialysis and patients with AKI. Adequate volume status is difficult to achieve due to the lack of established criteria for defining excess volume and a clear definition of “euvolemia” In patients with AKI requiring CRRT, the prescription CRRT dose, including the ultrafiltration rate, is typically influenced by each clinician’s considerations. This is troublesome because, if ultrafiltration is insufficient, persistent fluid overload occurs, jeopardizing organ function. However, excessive ultrafiltration for decongestion might result in iatrogenic hemodynamic instability and end-organ ischemia, especially in patients with AKI requiring CRRT. Bioimpedance integration into patient care may improve the objectivity of volume measurements and, for individuals receiving CRRT, the data presented in this study may serve as a practical guide for the safe and successful administration of ultrafiltration. As a limitation, our study acknowledges the potential interference of multiple devices on BIA measurements in critically ill ICU patients. Accurate measurements may require disconnecting or temporarily turning off certain devices, and the use of BIA should be implemented with consideration of the patient's clinical condition.

In conclusion, this retrospective analysis was conducted using a multicenter prospective cohort in which fluid status techniques were assessed in critically ill patients with acute kidney damage who had begun CRRT. It should not be forgotten that the ultimate purpose of medical AI for patients with CRRT is to assist clinicians in providing more effective CRRT therapy by providing them with an accurate assessment of a patient’s fluid status. Moving forward, it would be valuable to systematically validate the predictive potential and clinical usefulness of the AI models presented herein.

## Methods

### Study design, ethics statement, and participants

This multicenter cohort study presents an ML approach for the development cohort of the VolumE maNagement Under BCI in critically ill patientS on Continuous Renal Replacement Therapy (VENUS) trial. The detailed protocol of the VENUS trial has been previously described^37^. Briefly, the VENUS trial is a prospective, multicenter, randomized controlled trial (RCT) study on fluid management using BCM. The trial includes eight tertiary hospitals in Korea: Seoul National University Boramae Medical Center, Seoul National University Bundang Hospital, Seoul National University Hospital, Ewha Womans University Mokdong Hospital, Pusan National University Hospital, Kyungpook national university hospital, Hallym University Dongtan Sacred Heart Hospital, and Korea University Guro Hospital.

The present study is a retrospective analysis of the data from the trial for the purposes of using ML to evaluate the clinical value of the repeated monitoring of volume status in patients with AKI requiring CRRT on predicted prognostic outcomes. The study was approved independently from the VENUS trial by the institutional review boards of the aforementioned institutes for retrospective analysis. The following are the respective approval numbers of each hospital: Seoul National University Hospital Institutional Review Board (IRB No. B-1801/445–106), SMG-SNU Boramae Medical Center Institutional Review Board (IRB No. 20180108/10-2018-5/012), Seoul National University Hospital Institutional Review Board (IRB No. 1801-036-913), Ewha Womans University Medical Center Institutional Review Board (IRB No. EUMC 2018–01–071), Pusan National University Hospital Institutional Review Board (IRB No. H-1804-029-066), Institutional Review Board (IRB) of the Kyungpook National University Hospital (IRB No. KNUH 2020–01-034), Hallym University Dongtan Sacred Heart Hospital Institutional Review Board (IRB No. HDT 2020-01-011, and Korea University Guro Hospital Institutional Review Board (IRB No. 2020GR0197). The requirement for informed consent was waived by the IRB of (Seoul National University Hospital IRB, SMG-SNU Boramae Medical Center IRB, Seoul National University Hospital IRB, Ewha Womans University Medical Center IRB, Pusan National University Hospital IRB, Kyungpook National University Hospital IRB, Hallym University Dongtan Sacred Heart Hospital IRB, and Korea University Guro Hospital IRB owing to the retrospective design of the study. The study was performed in accordance with the principles of the Declaration of Helsinki, and clinical data from patients were obtained after receiving approval from the Institutional Review Board (IRB) at each center.

Patients with AKI requiring CRRT who were admitted to the ICU of the participating hospitals from November 2017 to November 2019 were included (Fig. 1). Patients were excluded if they were ≤ 18 years of age or had no mortality data.

### Clinical and laboratory evaluations

Demographic, clinical, and biochemical data were obtained immediately before CRRT initiation. Clinical data, including hemodynamic monitoring and laboratory data, were obtained on the same day before CRRT initiation. To evaluate adverse events, data on the number of hypotension episodes; usage, type, and dosage of vasopressors; and use and dose of diuretics were also collected. Moreover, data on complete blood cell counts, electrolytes, BUN, serum creatinine, albumin, aspartate aminotransferase, alanine aminotransferase, calcium, phosphorus, C-reactive protein, prothrombin time, and lactate levels were collected in the ICU. The variables contributing to AKI were originally classified and validated by the researchers using electronic medical records. The choice to provide vasopressors or mechanical ventilation was made by the attending doctors or intensivists based on the participants’ hemodynamic condition. The Acute Physiology and Chronic Health Evaluation (APACHE) II score^38^, SOFA score^39^, and the age-modified CCI were used as indicators of disease severity and comorbidities^40^.

The InBody S10 (InBody, Seoul, Korea)^28^, an impedance-based body fat analyzer, was used to measure fluid status at 0, 1, 2, and 7 days after CRRT initiation. TBW, intracellular water, extracellular water (ECW), segmental water, ECW/TBW values, fat-free mass, soft lean mass, segmental lean mass, body fat mass, and percent body fat were measured^41^. A bioimpedance analysis (BIA) showed differences in electrical conductivity according to the amount of water/electrolytes in each tissue. As BW passes an electrical current, the volume of BW can be obtained by measuring the resistance value obtained^41^. TBW/H^2^ and body weight were used as indicators of excess fluid balance.

### CRRT protocol

CRRT was performed using the PRISMA FLEX system (Gambro AB, Stockholm, Sweden) and AV1000 (Fresenius Medical Care, Bad Homburg, Germany). The prescribed CRRT dose varied from 35 to 65 mL/h/kg according to each patient’s clinical needs and attending physicians or intensivists’ decision. After initiating CRRT, the attending physicians and experienced nurses evaluated participants’ body weight, urine output, laboratory results, actual administered dosages, and hemodynamic status. These data were reviewed with the nephrologists to ensure that CRRT was adequate. The dialysate and replacement fluid ratio was maintained at 1:1. CRRT was administered using an internal jugular vein or femoral vein dual-lumen catheter. Unfractionated heparin or nafamostat mesilate was used as the anticoagulant. The CRRT maintenance period was based on each patient’s clinical status, with weaning from CRRT performed for any of the following reasons: systolic blood pressure > 120 mmHg and heart rate < 90/min, maintained without the use of vasoactive drugs; urine output maintained at ≥ 1000 mL, with or without the use of diuretics; renal recovery confirmed by the attending physician; and conventional hemodialysis (HD) deemed possible, even if renal function did not recover.

### Clinical outcomes

The primary outcome was in-hospital mortality. Secondary outcomes included the comparison of fluid balance by TBW/H^2^ and body weight between the event (death) and control (alive) groups. Events were defined as in-hospital mortality and RRT dependence among survivors at 90 days.

### Analysis using ML methods

We first introduced the traditional ML methods that we used as baseline methods for the experiments, along with an imputation method. Subsequently, a more advanced method, based on modern deep-learning architectures, was introduced. For extended methods related to modern deep learning, please refer to the included online supplemental data (see Supplementary Information).

### Statistical analysis

All statistical analyses were performed using the R statistical language (version 3.0.2, The Comprehensive R Archive Network: http://cran.r-project.org). The Multivariate Imputation by Chained Equations (MICE) package was used to compute missing values for continuous and categorical data. Python version 3.6.5 and TensorFlow 1.14.0 were used to implement the deep-learning models. Data were evaluated using the IBM SPSS software (version 22.0; IBM Corp., Armonk, NY, USA). Continuous variables of baseline characteristics and biochemical data are presented as mean ± standard deviation. Student’s t-test was used to test between-group differences in continuous variables, whereas the chi-squared test was used for categorical variables.

## Supplementary Information

Supplementary Information.

## Data Availability

All data associated with this study are available from the corresponding author upon reasonable request.
