# Supplementary material for: Predicting outcomes of continuous renal replacement therapy using body composition monitoring: a deep-learning approach
Source: Sci Rep. 2023 Mar 21;13:4605. doi: 10.1038/s41598-023-30074-4 (PMC10030803; doi:10.1038/s41598-023-30074-4)
Supplement: Supplementary file 1 — Supplementary Information. [file 41598_2023_30074_MOESM1_ESM.pdf]

[Title page]

Predicting Outcomes of Continuous Renal Replacement Therapy Using Body Composition Monitoring: A Deep-Learning Approach

Kyung Don Yoo[1*], Junhyug Noh[2*], Wonho Bae[3], Jung Nam An[4], Hyung Jung Oh[5], Harin Rhee[6], Eun Young Seong[6], Seon Ha Baek[7], Shin Young Ahn[8], Jang-Hee Cho[9], Dong Ki Kim[10,11,12], Dong-Ryeol Ryu[13], Sejoong Kim[11,14,15], Chun Soo Lim[11,12,16], and Jung Pyo Lee[11,12,16,¶], on behalf of Korean Association for the Study of Renal Anemia and Artificial Intelligence (KARAI)[17]

[1]Division of Nephrology, Department of Internal Medicine, Ulsan University Hospital, University of Ulsan College of Medicine, Ulsan, Republic of Korea

[2]Lawrence Livermore National Laboratory, California, United States

[3]University of British Columbia, Vancouver, Canada

[4]Division of Nephrology, Department of Internal Medicine, Hallym University Sacred Heart Hospital, Anyang, Republic of Korea

[5]Division of Nephrology, Department of Internal Medicine, Sheikh Khalifa Specialty Hospital, United Arab Emirates

[6]Division of Nephrology, Department of Internal Medicine, Pusan National University Hospital, Busan, Republic of Korea

[7]Division of Nephrology, Department of Internal Medicine, Hallym University Dongtan Sacred Heart Hospital, Hwaseong, Republic of Korea

[8]Division of Nephrology, Department of Internal Medicine, Korea University Guro Hospital, Seoul, Republic of Korea

[9]Division of Nephrology, Department of Internal Medicine, Kyungpook National University Hospital, Daegu, Republic of Korea

[10]Division of Nephrology, Department of Internal Medicine, Seoul National University Hospital, Seoul, Republic of Korea

[11]Department of Internal Medicine, Seoul National University College of Medicine, Seoul, Korea

[12]Kidney Research Institute, Seoul National University College of Medicine, Seoul, Korea

[13]Division of Nephrology, Department of Internal Medicine, School of Medicine, Ehwa Womans University, Seoul, Republic of Korea

[14]Division of Nephrology, Department of Internal Medicine, Seoul National University Bundang Hospital, Seongnam, Republic of Korea

[15]Center for Artificial Intelligence in Healthcare, Seoul National University Bundang Hospital, Seongnam, Republic of Korea

[16]Division of Nephrology, Department of Internal Medicine, Seoul National University Boramae Medical Center, Seoul, Republic of Korea

[17]Korean Association for the Study of Renal Anemia and Artificial Intelligence (KARAI), Anyang, Republic of Korea

*KD Yoo and J Noh contributed equally to the manuscript as lead authors.

¶Correspondence to:

Jung Pyo Lee, MD, PhD

Department of Internal Medicine, Seoul National University College of Medicine, Seoul National University Boramae Medical Center, 20 Boramae-ro 5-gil, Dongjak-gu, Seoul 156-707, Korea

Tel: +82-2-870-3206, Fax: +82-2-870-2826

E-mail: nephrolee@gmail.com

Table S1. Distribution and proportion of mortality according to CRRT delivered dose.

Delivered dose quartile	Q1	Q2	Q3	Q4	*P*-value
					0.398
Event (death) (n, %)	110 (56.4)	126 (63.0)	121 (64.0)	123 (62.8)	
Control (alive) (n, %)	85 (43.6)	74 (37.0)	68 (36.0)	73 (37.2)	
Delivered dose, mean (SD), mL/kg/h	24.75±3.50	31.13±1.29	35.73±1.45	45.07±7.89	<0.001

Table S2. Classification model for mortality using the conventional algorithm using setting 1.

Setting	Validation method	Test set size	Independence variables	Model	Test performance (AUC)
1	One validation set	234	68	Random forest	0.7655
1	Cross-validation	234	68	Random forest	**0.7637**
1	Cross-validation	234	68	Bagging	**0.7503**
1	One validation set	234	68	Bagging	0.7339
1	Cross-validation	234	68	Lasso	0.7210
1	Cross-validation	234	68	Ridge	0.7207
1	One validation set	234	68	Ridge	0.7127
1	One validation set	234	68	Lasso	0.7039
1	One validation set	234	68	Decision tree	0.6774
1		234	68	Logistic regression	**0.6770**
1	Cross-validation	234	68	Decision tree	0.6021
3		234	1	APACHE II score	**0.5646**
4		234	1	SOFA score	**0.6505**

AUC, area under the curve

The Table of Contents for Supplemental Methods

1. ***Analysis Using Machine Learning Methods***
2. ***Logistic regression***
3. ***Decision tree***
4. ***Neural network***
5. ***Treatment of missing values for machine learning approach***
6. ***Deep learning method: recurrent neural network with autoencoder imputation***

Analysis Using Machine Learning Methods

In this section, we first introduce the traditional machine learning methods that we use as baselines for the experiments along with an imputation method. Then, we propose a more advanced method based on modern deep learning architectures.

Logistic regression

One of the most popular machine-learning algorithms for classification tasks is logistic regression, which is one type of generalized linear models (GLM). Instead of assuming that a dependent variable is a normal distribution in the case of a linear regression model, it assumes that a dependent variable is a Bernoulli distribution. Hence, logistic regression converts a linear combination of independent variables to binary-valued outcomes using a logit function formulated as $\pi(X) = 1/(1 + \exp(-\beta X))$, where $\pi(X)$ indicates the probability of the dependent variable, y, being in class 1 given the independent variables, or simply p(y=1|X) [1]. A logistic regression model is trained to minimize a predefined cost function which, in our case, is defined as cost(y^, y) = sum –ylogy^ – (1–y) log (1–y^), where y^ is equivalent to p(y=1/X). To avert a problem of overfitting, which prevents a model from generalizing well on unseen data, we further apply Lasso and Ridge regularization to the cost function, which constrains the cost function using ||w||_1 and ||w||_2^2, respectively.

Decision tree

A decision tree algorithm, another commonly used classification algorithm, is simple and intuitive yet robust. It is eay to implement the decision tree algorithm, and interpret its results, compared to many other machine-learning methods. Even so, it is robust due to its nature of nonlinearity [2]. We employ a type of decision tree algorithms, called classification and regression tree (CART). CART forms a binary tree and gradually expands its leaf nodes to maximize purity measurement, or equivalently, minimize impurity measurement. Among three commonly used impurity measurements, we choose Gini index, which measures the impurity of internal nodes. The algorithm expands until it meets stopping rules specified as hyperparameters [2].

To further enhance the performance of machine learning methods, ensemble algorithms are often employed. Ensemble algorithms combine multiple base learners to improve the predictive performance more than a single base model. In this paper, we use bootstrap aggregating, also known as bagging [3], and random forest [4] as ensemble methods. Bagging consists of multiple base models independently trained on bootstrapped samples of the same size from the training dataset. In inference time, it averages the output predictions of the base models if a task is a regression problem whereas it selects the class with the most votes if a task is a classification problem. The random forest algorithm adds more randomness than bagging. It not only bootstraps samples but randomly chooses a fixed number of attributes among all the attributes available and finds the best split using them [5]. In this way, it improves the accuracy of the output predictions. We choose CART as a base learner for both bagging and random forest [2].

Neural network

A neural network is a network of neurons that aims to recognize underlying relationship of data through a process that imitates how a human brain operates. It consists of input, hidden, and output layers. An input layer corresponds to the variables of input data. Input data passed into a neural network through an input layer is passed into a hidden layer, which linearly combines the input data and modifies it using a nonlinear function, also known as an activation function. Then, the output of the hidden layer is passed into either the next hidden layer or output layer. In general, a neural network can be formulated as follows:

$$z_m = \sigma(\alpha_{0m} + \alpha_m{}^T x), \quad m = 1,2,\ldots,M$$
$$t_k = \beta_{0k} + \beta_k{}^T z, \quad k = 1,2,\ldots,K$$
$$f_k(x) = g_k(t)$$

where $z = (z_1,z_2,\ldots,z_M)^T$ is a hidden layer and $t=(t_1,t_2,\ldots,t_M)^T$ is an output layer. Also, $\sigma(\cdot)$ and $g(\cdot)$ are activation functions, which add nonlinearity to the inputs. Theoretically, a neural network can approximate any functions for both classification and regression problems. We design the architecture of a neural network to solve a binary classification problem.

A neural network is trained to minimize a loss function as a proxy to improve its performance of interest, which in our case, is classification accuracy. Although there are many options for a loss function, cross-entropy loss (defined below) is generally used for a classification task. Due to the nonconvexity of a neural network in terms of its parameters, it is not possible to compute a global minimizer analytically using convex optimization methods. Instead, numerical optimization methods, such as stochastic gradient descent, are used to find a local minimizer.

Treatment of missing values for machine learning approach

Multivariate Imputation by Chained Equation (MICE). The MICE, defined in statistical libraries in R (version R 3.4.4; The Comprehensive R Archive Network: http://cran.r-project.org), creates imputations for multivariate missing values of both continuous and categorical variables based on fully conditional specification where each missing value is sequentially imputed conditioned on other variables [5].

Deep learning method: recurrent neural network with autoencoder imputation

Although traditional machine learning methods work well in practice, many previous works in machine learning have demonstrated that their performance is limited compared to deep learning methods. Thus, we propose a more advanced and robust algorithm using deep learning architectures. As a result, we employ (i) a Recurrent Neural Network (RNN) such as Long Short-Term Memory (LSTM) to overcome time-sequential longitudinal observational

nature of data, and (ii) an Auto Encoder (AE) to better impute missing data.

(i) The first feature of the longitudinal observational cohort is the presence of time-variable attributes. The change of those attributes might have played an essential role in predicting the target variable. RNN is a type of a deep neural network, and each unit is sequentially connected [6]. This structure allows the network to model the dynamics of features at each time step considering the dependency of them, which the conventional feed-forward artificial neural network cannot capture. However, for vanilla RNN, gradients can be either vanished or exploded while training if the input sequence is long. This is called the problem of Long-Term Dependencies (LTD) [7]. To solve this problem, special instances of RNN such as LSTM, have been introduced. LSTM unit consists of an input gate, output gate, forget gate, and memory cell.

(ii) The second feature of the inevitable nature of the observational cohort is the existence of missing data. Perhaps the simplest way to handle missing data is to discard every data point containing at least one missing. However, this method can cause two major problems; statistical significance may be lost due to decrease in data size, and it may also introduce additional bias to a model due to the discrepancy with the population distribution. To solve these problems, we used an AE, a neural network that simply predicts the input value as an output value. If we set the number of nodes in the hidden layer to less than the input layer, AE can learn the compact representation of the input. This constraint enables us to learn how to express data efficiently, and it is possible to use this AE to express information including missing values. In the training process, some input values are removed randomly, and AE is trained to restore them as the original values. In the inference process, the encoded features of an input is used regardless of the existence of missing values.

Implementation and evaluation details

Regardless of method, we split our data into train (70%) and test (30%) sets for experiments. Due to the limited quantity of data, we perform 5-fold cross-validation to prevent our model from being overfitted. After the cross-validation, we evaluate our model using the test sets. We utilize widely used ML models (single models such as classification and regression trees, and logistic regression as well as ensemble models such as bagging and random forest) [2-4] as

baselines. We compare the performance of these methods using Area under the Curve (AUC) metric on a held-out test set.

REFERENCE

1. Dobson, A.J. An introduction to generalized linear models. *Journal of Statistical Planning and Inference* **32**, 418-420 (1992).
2. Breiman, L., Friedman, J., Stone, C.J., & Olshen, R.A. Classification and Regression Trees. *CRC press* (1984).
3. Breiman, L. Bagging Predictors. *Mach. Learn* **24**, 123-140 (1996).
4. Breiman, L. Random forests. *Mach. Learn* (2001).
5. Buuren, S., & Groothuis-Oudshoorn, K. Mice: Multivariate imputation by chained equations in R. *JSS.* **45**(2011).
6. Diez-Sanmartin, C. & Sarasa Cabezuelo, A. Application of Artificial Intelligence Techniques to Predict Survival in Kidney Transplantation: A Review. *J Clin Med* **9**(2020).
7. Burlacu, A.*, et al.* Using Artificial Intelligence Resources in Dialysis and Kidney Transplant Patients: A Literature Review. *BioMed research international* **2020**, 9867872 (2020).
